# A Significant Meeting

**Published:** 1915-08

**Authors:** 

**Affiliations:** San Antonio


### A Significant Meeting.

On May 26th there met at the Academy of Medicine, New York City, under the auspices of the American Society of Medical Sociology, a representative body of thinking men and women for the purpose of discussing the great question of family limitation. That such a meeting as this should be held and endorsed by some of our most progressive professional men and laymen, marks a decided change in the sentiment of our people. Indeed, nothing could more indicate the trend of progress toward the betterment of the human race. The "mores" of society are the most difficult barriers to any reform, and to see the strongest of these begin to be metamorphosized gives encouragement and hope to our evolutionary spirit.

The venerable Dr. A. Jacobi presided, and the following are his opening remarks as quoted from *The Critic and Guide:*

"Modern society is built on the family, the crude teachings of anarchists and socialistic teachings notwithstanding; a model society on a model family. This consists of a healthy father, mother and children.

"The future of mankind is conditioned by its children. Unless they be healthy and fit to work, physically and mentally, they cannot perform any duty in the service of the family, the commonwealth or the State. A large percentage of those born do not reach the average development required for that purpose. Hereditary influences propagate epilepsy, idiocy, feeble-mindedness and cretinism. They should not have been permitted to be born. Many of our States have passed laws to prevent the propagation of such individuals by sterilizing persons, mainly men, by performing a kind of operation. That the subject has caused a fair amount of literature to be produced not only in America but in Germany was not to be wondered at. Psychiatrists, forensic lawyers, jurists and doctors have been interested. Syphilis is or may be transmitted one or more generations. That is why health departments and many clergymen exert themselves to prevent the marriage of persons so diseased. Nobody finds fault with that. Tuberculosis may be transmitted bodily, but it is more often transmitted by disposing the newly born to be feeble and amenable to disease of any kind, and constitutionally infirm. The doctors forbid persons with tuberculosis to get married, and correctly so.

"One of the great social drawbacks is poverty. It includes overwork for men and women, improper, insufficient, or irregular feeding, coarse or insufficient clothing, tenement dwellings cold or overheated and wet, congestion and want of air, temptation, dissipation, neglect of children, too many children, much disease, many deaths. Even these deaths are expensive, break into scanty savings, and increase poverty. Would it be wise on the part of the children not to be born? Surely. But here they are, born for starvation, or factory work, or prostitution, or an emperor's war game.

"Born they are and United States or State laws see to it that whoever advises that they must not be born, to prevent them being born without any danger or harm to father or mother, is branded a criminal. The prohibition of unnecessary and not wanted accessions of human beings is considered criminal. As it is true that every nation has its ideals, so every nation has its own idiosyncrasies. What in Europe is right by law and carried out by scientific studies, is forbidden among us. Even worse than that is the fact that what is stamped as a crime today will be considered a beneficial measure tomorrow, and vice versa.

"Outside of poverty, look at the rich, the well to do or the middle class. Yesterday you could read in the morning papers the report coming from a famous girls' college. In twenty years not one-half of the graduates were married; those who were had one and one-half child to one wedlock, only one and one-half. Those that make the laws or those who execute them do not charge them with race suicide. We quietly look at the extinction of that class of people who came on the Mayflower.

"Consider the middle class family. If there are too many children for comfort and health and life, the family will sink back into want and poverty. If there were fewer that family would exactly be what you want it to be, the prop and staff of the State. But if you, a statesman or a physician, advise them how to avoid poverty and the level of the proletariat, you run the risk of falling into the hands of spies and detectives. Several have tried their hands on me this week. That is, so I understood, why we are here. Speakers will proclaim to you briefly that the prevention of uninvited or injurious births must be permitted or even advised. It will be the domain of the physician to teach and advise in what way men and women in wedlock may regulate their own family, their sizes and ages. That is what this meeting is to proclaim, no longer to study. No time can be given for that. There will be no discussion here, and none tonight. What you have come here for is to demonstrate that you feel you are right, that you are a nucleus, around which democratic men and women may conglomerate into a movement for liberalization of views and a generous law. In a year or two public opinion will veer about and people will wonder how stupid and callous and ungenerous they have been. Your speakers have made up their minds to confine themselves to a few minutes only and that is why your chairman may hold them to their self-imposed limits."

